# Nutritional Issues Faced by Patients with Intestinal Stoma: A Narrative Review

**DOI:** 10.3390/jcm12020510

**Published:** 2023-01-08

**Authors:** Izabela Michońska, Ewelina Polak-Szczybyło, Aneta Sokal, Sara Jarmakiewicz-Czaja, Agnieszka Ewa Stępień, Katarzyna Dereń

**Affiliations:** Institute of Health Sciences, College of Medical Sciences, University of Rzeszow, 35-959 Rzeszow, Poland

**Keywords:** diet, ileostomy, colostomy, stoma, nutrition

## Abstract

The incidences of colorectal cancer and inflammatory bowel diseases are increasing in the developed countries of Western Europe and North America, and consequently, the prevalence rate of temporary or permanent stomas has increased in recent years. Nevertheless, the amount of research in the nutrition field in the case of patients with stoma is rather limited. This review article aims to assess the impact of nutrition on an intestinal stoma and possible sequelae resolved with nutritional therapy. The research analyses conducted thus far indicate a significantly increased risk of developing malnutrition, vitamin deficiency (especially group B), and an increased number of discharges from the stoma and its relevance with abnormalities found in electrolyte concentrations.

## 1. Introduction

The stoma serves in moments, whereas for various causes the excretion of faeces is impossible physiologically [1]. The term intestinal stoma might be used interchangeably with an artificial rectum. This review deals with excretory intestinal stomas, but does not discuss enteral feeding, i.e., gastrostomies and jejunostomies [1,2]. There are many research difficulties regarding studies related to the nutrition of stoma patients due to the wide range of different patients. The medical indications to perform an excretory stoma are mainly neoplastic diseases, especially those affecting the distal parts of the gastrointestinal tract, such as the large intestine. Other common indications are congenital anomalies and inflammatory bowel diseases (IBD) such as Crohn’s disease (CD) and ulcerative colitis (UC) [1,3,4,5,6,7]. A Swedish population-based study showed that even with the increasing use of anti-TNF (tumor necrosis factor) drugs and low rates of proctectomy, the total number of stomas removed in patients with a diagnosis of CD, within 5 years of diagnosis, did not decrease from 2003 to 2019 [4]. Intestinal stomas placed pro tempore decompress the diseased part of the intestine for the duration of the treatment. After eliminating the cause of the illness, the continuity of the digestive system is restored, unless there are contraindications [1,3]. Both temporary and permanent intestinal stoma require changes in the diet due to the emergence of disparate gastroenterological problems. Partially altered bowel function and underlying pathology expose patients to numerous nutritional deficiencies, including malnutrition.

This narrative review aims to analyse the scientific reports on the nutrition problems of patients with an intestinal stoma. This review discusses nutrition-related complications among patients with a stoma, their relationship to the stoma site, and measures to cope with it. In addition, this review assesses the occurrence of malnutrition in patients with an intestinal stoma, the risk of developing nutritional deficiencies, and the supply of macro- and micronutrients.

## 2. Methods

Internet databases (including Medline, PubMed, ResearchGate) were searched for review materials. The following terms were used: proctocolectomy, stoma, ileostomy, colostomy, nutrition and diet (October 2022). Inclusion criteria: articles in English; articles about ileostomy and colostomy, research with adult patients, meta-analyses, systematic reviews, clinical studies, including randomized controlled trials, observational studies, historical data and reviews. Exclusion criteria: articles in a language other than English, articles about urostomy, animal studies, research with child and adolescent (<18 years old), case studies, scoping reviews, narrative reviews, articles on microbiota and probiotic therapy. The papers dealt with in the main part of the article, that is, nutritional issues, and were published between 2001 and 2022.

## 3. Nutrition Issues

### 3.1. The Risk of Developing Malnutrition or Nutrient Deficiencies in Patients with a Stoma and How to Monitor Malnutrition in Surgical Patients

#### 3.1.1. Pre-Operative Period and Tools for Diagnosis of Malnutrition at Different Stages (Patient Preparation and Hospital Care of Surgical Patients, including Those with a Stoma)

Not only a nutritional intervention after the stoma creation is crucial but also the nutritional status of the patient before the surgery. Fulham et al. [8] also emphasized that the nutritional status before the stoma surgery, as well as the patient’s age and the cause of the main disease, are of immense importance. Arnott et al. [9] in their study published in 2022 also considered the importance of patient nutrition before surgery to restore the continuity of the gastrointestinal tract. The researchers report that a considerable number of patients undergoing Hartman reversal (HR), were identified as being at substantial risk for malnutrition. Poor nutrition was associated with worse postoperative outcomes, including wound infection or patient death. The cited team of researchers supports the use of preoperative screening for malnutrition. Such screening measures can make it possible not only to diagnose malnutrition among patients, but more importantly to take appropriate steps to improve the patient’s nutritional status and avoid complications [9].

In 2001, the Audit Commission and in 2003 the British Association for Parenteral and Enteral Nutrition (BAPEN) highlighted the importance of screening in the context of malnutrition, both upon admission to the hospital and regularly during the entire stay [10]. In 2018, the Global Leadership Initiative on Malnutrition (GLIM) issued unified criteria for the diagnosis of malnutrition, which may also be efficient for patients with an ostomy. The main criteria suggested by participants are non-volitional weight loss, low body mass index (BMI), reduced muscle mass, reduced food intake or assimilation and disease burden or inflammation. As diagnostic tools, in the case of malnutrition, GLIM recommends the use of the following scales: NRS-2002 (Nutritional Risk Screening 2002), MNA-SF (Mini Nutritional Assessment—Short Form), MUST (Malnutrition Universal Screening Tool), SGA (Subjective Global Assessment), and many others [11].

There are no recommendations for the nutrition assessment tool considering the nutritional status in both the ileostomy and the colostomy groups. Usually, universal questionnaires such as MUST are used. MUST is widely used in hospitals to predict the length of stay and mortality. According to the authors, the tool can help diagnose malnutrition such as being overweight and obesity, and most importantly, it can be used even in patients with fluid disturbances. The MUST questionnaire permits for a quick assessment of the patients’ risk of malnutrition, as it consists of only five steps: BMI assessment, evaluation of casual loss of body weight in the last 3–6 months, and the presence and possible impact of acute disease on the patient’s nutritional status. In the last two steps, the points are added up, and according to the level of risk, appropriate dietary guidance is issued [10]. However, its modified version, the Perioperative Nutrition Screen (PONS) is recommended by Wischmeyer et al. [12] for assessing the nutritional status of patients after a gastrointestinal surgery. PONS is another short questionnaire that indicates the need for preoperative nutrition, and clinical or nutritional intervention. Patients are qualified in case of at least one of the following occurs: BMI < 18.5 kg/m^2^ or <20.0 kg/m^2^ (age > 65 years), recent weight loss and reduced half appetite at the very least. An additional condition related to the surgical risk is the decrease of the albumin level below 3.0 g/dL [12].

Santamaria et al. [13] used the SGA for the assessment of nutrition in a group of patients with ileostomy with High Output Stoma (HOS), which is a well-known and standardized tool. In addition, patients with colon cancer who underwent a scheduled ileostomy surgery, were diagnosed before and after with the MNA [14]. The last two questionnaires are based on similar questions, and are slightly longer, however, still fit on one page. This allows them to be carried out efficiently and repeated regularly during the patients’ stay in the hospital [13,14,15]. If a patient is qualified for a nutritional treatment, the first step is to perform an in-depth evaluation of the nutritional status of that patient. The next step is the qualification to the selected type of nutritional intervention. Overall, an in-depth assessment of the patient’s condition is performed by a nutritional interview, anthropometric measurements, and biochemical and immunological tests [8,16]. The body mass index, also known as the Quetelet II index, is most often used to assess body weight. Other commonly used measures are the thickness of the fat-dermal fold above the triceps muscle of the non-dominant arm (triceps skinfold thickness, TST), mid-arm circumference (MAC), and whole-body bioelectrical impedance analysis (BIA) [10].

#### 3.1.2. Immediate Post-Operative Period

**Figure 1:**
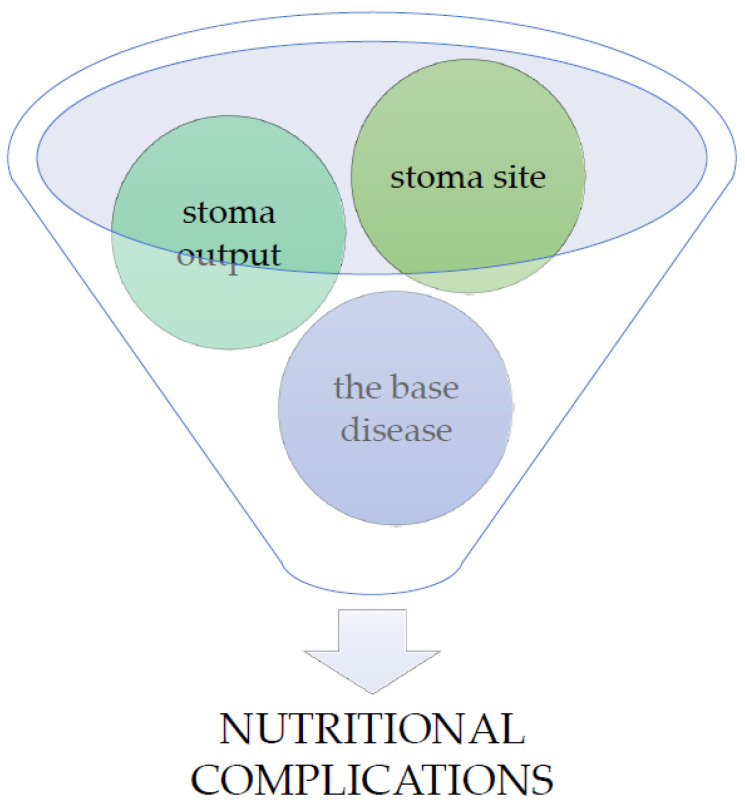
Major situations affecting nutritional complications in stoma patients.

In the initial period, after the surgery and during convalescence, the greatest attention is paid to the physical and psychological aspects of preparing the patient for a life with an ostomy [17]. Therefore, it is easy to overlook the nutritional needs resulting from comorbidities such as cancer or diseases of the gastrointestinal tract, which may exacerbate nutrient deficiencies or weight loss. Malnutrition is a condition that very often accompanies patients devastated by cancer or with intestinal diseases. It results from malabsorption and long-term hospitalization [18,19]. Nutritional complications are usually caused by stoma output, stoma site and base disease (Figure 1).

According to Fulham et al. [17], malnutrition in patients with impaired bowel function may be caused, in part, by the hospital routine. Meal delivery times are fixed and adapted to the needs of the serving staff and catering companies. The needs of patients with an ostomy are different. The consumption requirements concern smaller and more frequent meals, and more frequent intake of fluids during the main meals [17].

An observational study conducted by Mukhopadhyay et al. [16] involving patients with a created ileostomy demonstrated that early implementation of balanced oral feeding was associated with normal nutritional status. Bodyweight decrease and serum hemoglobin levels were noticed after one week of oral feeding. However, baseline biochemical (hemoglobin, albumin) and anthropometric (body weight) parameters were normal at the twelfth week of a follow-up [16].

#### 3.1.3. Delayed Post-Operative Period

Due to the physiology of the gastrointestinal tract, nutritional deficiencies may affect patients to a greater or lesser extent, depending on the location of the stoma creation. People with an ileostomy are more prone to nutritional deficiencies because most of the digestive system is served temporarily or permanently, and henceforth the mechanism of nutrient absorption is disturbed [8,15]. The sites of absorption of individual nutrients are shown in Table 1 [15].

### 3.2. Ileostomy

The small intestine is responsible for the absorption of most nutrients, including amino acids, carbohydrates, fats, minerals, and water [15]. When an ileostomy is established in the initial section of the ileum, the surface area of nutrient absorption may be significantly reduced. In such a case, the efficiency of the process may be impaired due to a reduced number of intestinal villi and microvilli [8,15].

When the intestinal stoma is created within the small bowel, the patient should be monitored for the consumption of fat and of fat-soluble vitamins such as A, D, E, and K. It is also worth paying attention to the level of vitamin B12 (cobalamin), which is absorbed in the ileum, in the presence of Castle’s intrinsic factor. In the case of an ileostomy, when the entire colon is absent or not in continuity, the patient has also been deprived of the endogenous source of vitamins and nutrients produced by the gut microbiota. These nutrients are vitamins: K, B, folic acid, and short-chain fatty acids (SCFA) [8,15]. The study by de Oliveira et al. [20] showed that in patients with an ileostomy, low absorption of fat and niacin occurs much more often than in those with a colostomy.

A stoma in the small intestine predisposes patients to an increased amount of water excreted due to the lack of reabsorption in the large intestine. Patients with an ileostomy most often pass stools in a liquid or semi-liquid form. If the stoma is in the proximal part of the small intestine, in addition to absorbing water, there may be problems with the absorption of sodium and potassium. The remaining nutrients should be absorbed normally if no inflammation develops in the intestine and there is no damage to the intestinal villi [8,15].

In the scientific literature on stomas and nutrition, authors pay a lot of attention to the three complications: excessive amount of discharge in the stoma bag, disturbances in the water and electrolyte balance, and leakage of intestinal contents in the stoma area [21,22,23]. Arenas-Villafranca et al. [22] found that the presence of a high-output stoma produces an increased amount of fluid (>1500/2000 mL/day) and is associated with electrolyte disturbances. Study results outlined that all HOS cases were associated with an ileostomy and resulted in an extension of the hospital stay by 6 days compared to patients without HOS [22]. De Oliveira et al. [20] proved that the percentage of patients avoiding the consumption of selected groups of food products is significantly higher among patients with an ileostomy. According to the authors, adjusting the diet to one’s own needs and excluding certain foods from it may also increase the risk of malnutrition in patients with a stoma in the small intestine [20].

### 3.3. Colostomy

The responsibility of the water and electrolyte absorption is covered by the large intestine; thus, sodium and potassium are the most essential elements. In people with a preserved large intestine, water is recovered from the contents of the intestine along its entire length. The body recovers water even from the formed stool—this mechanism causes constipation if the stool is held for too long. The second function, apart from water absorption, is the uptake of sodium in this section of the gastrointestinal tract. It occurs in the colon and helps maintain the body’s water and electrolyte balance. In the case of partial or complete exclusion of the colon, patients often develop electrolyte disturbances [8,15].

The risk of nutritional deficiency is greater when the colostomy is created due to inflammatory bowel disease (CD or UC). However, it is related to the underlying disease, especially during its exacerbation. In both cases, there is a malabsorption of proteins and the medications used can lead to a deficiency of calcium, potassium, and zinc. Frequently, deficiencies of fat-soluble vitamins, folic acid, vitamin B12, as well as the minerals calcium, magnesium, and zinc occur in patients with CD as a result of extensive inflammation or removal of a part of the intestine [21,24]. Due to bleeding, patients with ulcerative colitis tend to lose iron, fluid, and electrolytes [25]. Chandramouli et al. [26] indicate that malnutrition may be one of the complications of colostomy. Furthermore, they indicate that transverse colostomy had a lower rate of malnutrition than sigmoidal colostomy.

### 3.4. Demand for Energy, Macronutrients, Vitamins, and Micronutrients

There is no scientific data on the validity of increasing or not increasing energy supply in patients with an ostomy. Such data could be helpful, especially in patients who are malnourished or at risk of malnutrition, as well as in those who suffer from peristomal complications related to nutrition.

De Oliveira et al. [20], in a study evaluating the consumption of energy and nutrients, did not find any deterioration in the nutritional status in both ileostomy and colostomy patients. On the other hand, the average usual energy consumption in the group of patients with an ileostomy was 20% lower than in patients with a colostomy.

In the study by Migdanis et al. [27], a decrease in the average energy consumption among patients with an ileostomy was reported to be approximately 100 kcal compared to the preoperative period. There were no such changes in the control group. In addition, patients with an ileostomy also lost weight and body fat percentage during this period. However, the authors emphasize that these data should be carefully applied to the general population with an ileostomy, as patients were examined in the period immediately after the surgery (40 days after the surgery). These people often eat food selectively for fear of an increased stoma secretion and leakage [27].

Nutrient absorption can be impaired in patients with ostomies; therefore, energy intake may increase to about 30% for inpatient with HOS [23]. Increasing the calorific value of the diet can be achieved by dietary modification or by using preparations from the ONS group.

The topic of energy loss is closely related to the topic of fat supply. Several years ago, clinicians recommended vast restrictions on fat intake to reduce the fatty diarrhoea that often-accompanied patients, especially those with an ileostomy. For the time being, very low-fat diets are not recommended because low fat consumption could relevantly reduce the caloric value of the diet [23]. Such restrictions can aggravate the problems of undernourishment or low weight in people and patients trying to regain lost weight. Additionally, fat deficit exposes the patient to a deteriorated absorption of fat-soluble vitamins [8]. It is recommended to limit saturated fats in favour of unsaturated fats, which are a source of phytosterols as well [28,29,30]. No strict guidelines have been found regarding the percentage of this macronutrient in the diet of a patient with a stoma. Each afflicted should be treated individually, and the fat supply should be appropriately matched to their current condition and the condition of the digestive tract.

The total amount of carbohydrates is generally not restricted, with a recommendation to ingest 40–50% of calories as carbohydrates. However, the type of component ingested is important. [23]. Although, the more valid issue seems to concern the quality and the gradual implementation of soluble fibre. Albeit the amount of fibre in the diet is a matter of individual consideration and depends on the type of stoma or the time passed since the surgery [8,23]. The basic products recommended for patients with a stoma are starch products such as potatoes, white rice, refined flour pasta or white bread [8]. In patients with ileostomy, suffering from dehydration or HOS often occurs, ergo it is recommended to avoid hypotonic fluids like sweet fruit juices or hypertonics like soft drinks and other sugar-sweetened beverages, which may worsen these problems [31]. For the same reasons, patients with HOS are also not recommended to consume sweeteners and products such as chewing gum [23]. Ingestion of a low-FODMAP (fermentable oligo-, di-, monosaccharides and polyols) diet may improve gastrointestinal symptoms and hydration in patients with HOS [32]. This diet, especially in the initial stages, is very restrictive and should be carried out under the supervision of a dietitian in order to ensure that nutrient intake is adequate and balanced [23,32]. On the other hand, among patients with colostomy whose gastrointestinal passage is less altered, the preventive use of foods with higher fibre content, such as brown rice or whole wheat bread, may be helpful. This may help to avoid constipation problems that accompany a significant percentage of patients with this type of stoma [8].

The usual supply of protein among patients with ostomies is recommended at the level of 20%, unless there are indications to increase its amount, such as convalescence or malnutrition [8,23]. Burch et al. [33] recommend that ostomies consume two to three servings of protein per day. Due to the limitation of the supply of saturated fat, lean meat, e.g., poultry, fish, eggs or lean dairy products are considered the preferred sources of protein. Despite wherethrough high content of insoluble fibre fractions and the bloating nature, the inclusion of legume seeds should be approached with caution, especially among patients with ileostomies. Protein is a macronutrient relatively well tolerated by both patients with an ileostomy and a colostomy; therefore, all individuals with a stoma are advised to take it daily in appropriately selected portions [8,23]. It should be remembered that, besides the protein content, dairy is also a reliable source of calcium, which may protect the patient against osteoporotic changes [33].

The scientific literature reports an increased risk of osteomalacia and osteoporosis, combined with a calcium deficiency in people who have an intestinal stoma created as a result of inflammatory bowel disease. There is a need for further research and guidelines from scientific societies in this regard because preventive measures can bring many benefits to the examined [34,35].

Unfortunately, thus far none of the global associations dealing with clinical nutrition have developed guidelines for the supplementation of vitamins or macro- and micronutrients in patients with a stoma. In the scientific literature, however, there are references to the risk of cobalamin and a vitamin K2 deficiency in people who have an ileostomy or a colostomy. This is due to these two vitamins being absorbed in the terminal ileum and colon, and not in the duodenum and jejunum, where most of the vitamins and minerals are absorbed [36]. No scientific papers directly addressing the subject of supplementation have been found, however, in one of the cross-sectional studies involving patients with an intestinal stoma. Schiergens et al. [7] associated vitamin B12, iron, and zinc deficiencies with a reduced quality of life. The study found an association between the lack of these nutrients and physical well-being and a reduced quality of life related to the gastrointestinal tract, measured by the Gastrointestinal Quality of Life Index (GIQLI). The strongest correlation was found between vitamin B12 deficiencies and the mental health of patients [7].

**Figure 2:**
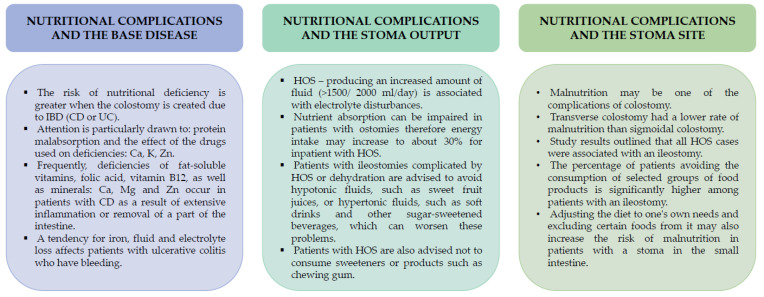
Key measures on the relationship between nutritional complications and stoma output, stoma cite and base disease.

It is worth emphasizing that the distribution of macro- and micronutrients may depend to a considerable extent on the underlying disease and the stages of its exacerbation or remission. The individual condition of the patient and the complications associated with the procedure or maintenance of the stoma will also be meaningful. In Figure 2, we have summarized the key measures of the relationship between nutritional complications and stoma output, stoma site and underlying disease (Figure 2).

### 3.5. Recommendations to Prevent Complications and Deficiencies

A balanced diet and properly conducted nutritional education perform a key role among the methods of preventing peristomal complications and deficiencies that may accompany patients [8,23,33,37].

A patient with an ileostomy is encouraged to chew their food thoroughly and to introduce fibre into their diet gradually after the creation of the ileostomy. These interventions can reduce common ailments such as diarrhoea, bloat, and odour as wells as intestinal obstruction. Patients with ileostomies can easily experience dehydration and electrolyte disturbances; thus, education on dehydration symptoms and treatment is important. [8,37]. In the past, men with dehydration with an ileostomy were advised to add a teaspoon of salt to drinks or to consume additional salty snacks, such as crisps, between meals. Currently, due to the high degree of processing of many food products (cold cuts, cheese and bread), and thus the relatively high salt content in the daily diet, this method is not recommended [8,33]. In line with the principles of a healthy diet, such as the nutritional pattern of the Dietary Approaches to Stop Hypertension (DASH) diet, all people should be careful about additional salt intake. Patients with HOS should be provided with adequate sodium rehydration solutions prepared with the use of salt in strictly defined proportions. On the other hand, patients with an ileostomy who do not have this problem, and patients with a colostomy should follow the principles of a healthy diet, e.g., not overdoing the supply of salt, monitoring for signs of dehydration and checking sodium levels prophylactically [23,38]. Pedersen et al. [39] report that in patients with an ileostomy, a single spot urine sodium sample taken in the morning to noon hours can reliably estimate the 24-h urine sodium excretion. Testing this in people with an ileostomy can, therefore, quickly identify a sodium deficiency and the sodium supply can be increased, if needed [39]. According to Medlin et al. [23] and Mountford et al. [31], patients with HOS and dehydration who frequently experience electrolyte disturbances should avoid excessive intake of hypotonic drinks (e.g., water, tea, and coffee limited to 500 to 1500 mL/day). While these hypotonic free water liquids are recommended for hydration of healthy individuals, these solutions may aggravate dehydration in patients with an ileostomy in whom sodium cannot be properly absorbed [23,31]. Instead, it is recommended for patients with a stoma to consume a rehydration solution consisting of 1000 mL of water, 20 g of glucose, 2.5 g of sodium bicarbonate, and 3.5 g of sodium chloride (table salt) [23,31]. According to Kelly et al. [40], the use of appropriate rehydration solutions improves fluid absorption (↑ 60%) and sodium absorption (↑ 40%) in the jejunum and may improve the ileal absorption of these substances (↑ 20–30%) [40]. In patients with an ileostomy, hydration status assessment is recommended, and it may be based on urine urea and electrolyte levels, but the monitoring of urine sodium is a better option. Urine sodium, especially in patients with HOS, is recommended to be monitored every 2–3 months [23].

Other dietary interventions depend on the specific problem the patient is facing. In patients with bile acid stagnation, it is recommended to use oat bran with native β-glucans to increase their excretion. A study by Ellegård et al. [28] confirmed an improvement in bile acid metabolism following this dietary intervention. It was manifested in the increased excretion of 7α-hydroxy-4-cholesten-3-one, which was an intermediate in the synthesis of bile acids from cholesterol [28]. As a result, two other papers by these authors investigated the effect of plant phytosterols on patients with an ileostomy. The first study concerned the restriction of saturated fatty acids and increased supply of dietary fibre, while the second study concerned plant sterols derived from rapeseed oil and olive oil [29,30]. It was noticed that patients using food rich in phytosterols were characterized by an increased cholesterol excretion. This relationship is presumably due to the competition of phytosterols for a place in micelles with cholesterol from animal products [29]. It was also emphasized that rapeseed oil tends to reduce cholesterol absorption, increase the excretion of cholesterol and bile acids, and lower serum cholesterol compared to olive oil. The authors of the study suggest that this may be due to the difference in the concentrations of natural plant sterols occurring in these products [30].

Patients with an ileostomy will often find substantial amounts of fibre in the form of raw or unpeeled vegetables and fruits as the cause of some gastrointestinal problems. However, for people with a colostomy, these products can be immensely helpful in preventing constipation. Additionally, a varied diet rich in fruit and vegetables may also protect against vitamin and mineral deficiencies [33]. Arenas-Villafranca et al. [22] warn patients with a stoma against water-insoluble fibre fractions that may lead to intestinal obstruction. At the same time, they state that there is a small amount of good-quality clinical trials on soluble fibre in the nutrition of patients with a stoma [22].

**Figure 3:**
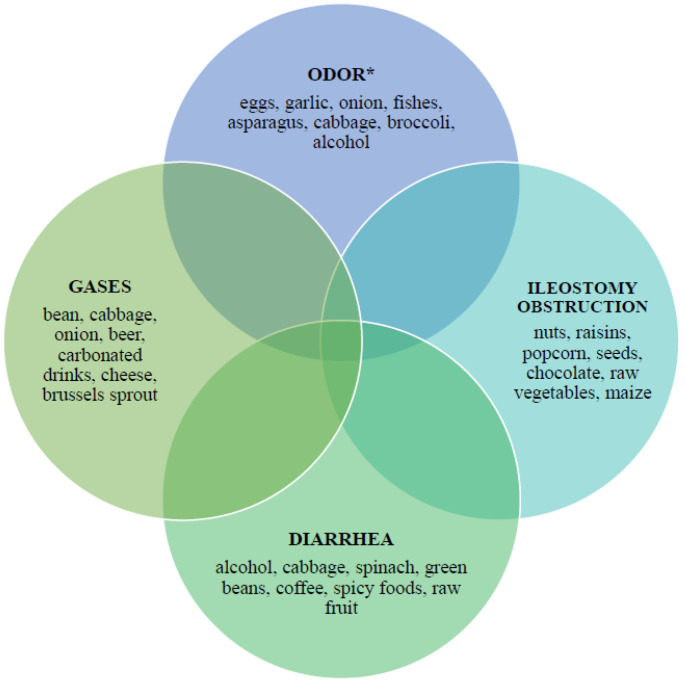
Foods that can cause selected types of digestive tract complications in patients with a stoma [41]. * Products that can reduce unpleasant odor: cranberry juice, parsley, buttermilk and yoghurt.

Gastrointestinal problems in patients with ostomies may be triggered by the ingestion of gas-causing foods, carbonated or sweetened beverages, or inadequate fluid intake. In addition, ingestion of some foods such as nuts, corn, beans, coconut, dried fruit, and mushrooms can lead to a mechanical blockage of the stoma. Consuming expired or improperly stored food carries the risk of microbial contamination [41]. Figure 3 summarizes the foods that may cause the most common gastrointestinal problems in patients with ostomies (Figure 3).

### 3.6. Nutrition Consultation and Education as a Tool for Effective Protection against Complications

Recently, some interesting studies on educational support and nutritional consultation for patients with an intestinal stoma have been published [13,42,43].

In a Spanish cohort conducted by Santamaria et al. [13] involving 170 patients (85 each in the study and control groups), patients underwent two nutritional consultations after hospital discharge. They found that such consultations significantly reduced the rate of hospital readmissions associated with high-secreting stoma and dehydration, which also resulted in significant savings for the hospital [13]. Other researchers have reached similar conclusions, citing HOS as the most common cause of readmission [37,38,39]. Many researchers reported significantly lower rates of patients returning to the hospital after implementation of a follow-up nutritional consultation compared to the non-intervention group in a retrospective study compared to other researchers [13,43,44,45].

In addition, Qu et al. [46] in a study published this year, reported that a combination of a nutritious meal, online advertising and education on the postoperative nutritional and psychological status of patients with a colostomy emerged due to cancer. Patients in the group undergoing the described intervention achieved statistically significantly better scores on indicators related to nutrition, immunological exponents or the WHO Quality of Life Assessment Test (WHOQOL—BREF) score. On the other hand, the scores of the self-rating anxiety scale (SAS) and self-rating depression scale (SDS), as well as the overall incidence of complications were statistically significantly lower in the intervention group [46].

Fernández-Gálvez et al. [42] subjected 253 ileostomy patients (117 in the control group and 136 in the study group) to a nutritional intervention based on the principles of the Mediterranean diet. In addition, patients were provided with nutritional counselling during periods of the reintroduction of oral nutrition, hospital discharge and the first follow-up visit. Patients in the study group mostly reported weight gain (positive effect), a decrease in dehydration, and a lower rate of re-hospitalization for stoma complications. The dietary model and nutritional consultation introduced made it easier for patients to eat a minimum of five meals a day and reduced their doubts or concerns about diet [42].

### 3.7. Basic Nutritional Guidelines for an Intestinal Stoma

At the outset, it should be noted that the diet of a person with a stoma is also dependent on their underlying pathology and may require the use of a given eating pattern or specific nutritional supplements, or the elimination of a particular group of products [16].

The diet of a patient with a stoma is gradually expanded, and a period of a few weeks is often taken as a return to the habitual diet. Nutrition in the postoperative period begins with a liquid and semi-liquid diet, gradually moving to an easily digestible diet with the limitation of nutrients such as fibre, fat or simple carbohydrates [47,48]. It is recommended for new products to be implemented into the diet individually and in tiny amounts. Thanks to this, when any problem occurs, it is easy to eliminate a product that caused them. After the initial recovery period, a nutrition style consistent with principles of a healthy and varied diet is recommended for patients with ostomies [14,45,48].

In the study of Toledano et al. [49], the impact of the time at which oral nutrition was introduced after the procedure of the stoma creation on the patient’s postoperative results was assessed. The previous and mostly purely theoretical post-surgical nutritional protocols suggested that oral food and fluids should be withdrawn to reduce the risk of complications [48,49]. The conclusions represented by Toledano et al. [48] stated that the faster introduction of an oral diet after the procedure resulted in a shorter time of first bloating, faster excretion of the first stool and a shorter time to restoration of the proper functioning of the gastrointestinal tract. The main limitation of the study was its retrospective nature and the fact that most patients who received oral nutrition for a shorter period of time were patients who underwent laparoscopic surgery, which is not always feasible. In addition, nutritional progress in patients was measured on a daily, not hourly scale, which made it difficult to capture more accurate data and perform a more detailed statistical analyses [48]. On the other hand, the team led by Petrelli [50] indicated that early oral feeding is safe and applicable in patients with a history of colorectal cancer and colectomy. The reasons for the failure of such an intervention were intraoperative blood loss and a too large volume of the expander used.

The general guidelines for ostomy patients emphasize the importance of regular meals, consuming plenty of fluids, and thoroughly chewing food to avoid stagnation in the gastrointestinal tract. Patients with a stoma are also recommended to consult a stoma nurse and a dietitian at every stage of nutritional management or in case of doubts [41].

## 4. Limitations

A limitation of this review may certainly be the relatively small number of clinical trials conducted with patients with an intestinal stoma removed for dietary nutrition or nutritional treatment. The dietary strategies used were also often heterogeneous, and the study groups of patients cited in randomized clinical trials were often few in number. Many of the studies on intestinal stoma patients are observational: cohort or retrospective studies. In summary, scientific papers on the issue of feeding patients with intestinal stomas are few, and often papers with a very low level of evidence.

Some of the cited studies also administered nutrition concurrently with medications, which may not fully reflect the actual situation regarding dietary nutrition for patients after an intestinal stoma procedure or who are in recovery. Few studies have also considered the nutritional status of the patient before the intestinal stoma procedure; however, sometimes this was impossible due to the unplanned nature of the operation.

As authors, we tried to make the studies we collected as objective as possible, which may be indicated by, among other things, clear inclusion and exclusion criteria for studies in the review, as well as the presentation of all study results, not just those supporting the hypothesis of a beneficial effect of nutrition on the patient.

Nevertheless, despite the authors’ efforts, a narrative review is not as objective a form of scientific work as a systematic review or meta-analysis of scientific studies. In contrast, these more highly regarded standards for synthesizing scientific evidence tend to focus only on a narrow area of an issue, are very time-consuming and require similar research criteria and conditions. In the field of the issue of feeding patients with an intestinal stoma, due to the small number of studies, the widely varying research conditions in the interventions used, or the stomas being placed on different segments of the intestine (small or large), it would be a very complicated or even impossible task to conduct a systematic review or meta-analysis.

There is a need for studies involving patients who have had an intestinal stoma removed for distinct reasons. Future studies should also standardize research conditions and think about conducting multicenter studies. It would also be necessary to look closely at the effect of the patient’s nutritional status before surgery and the impact of this factor on improving the prognosis for patients with an intestinal stoma.

## 5. Conclusions

Progress in stoma care has improved the prognosis and quality of life for many patients worldwide. However, they also revealed the essential role to provide professional care by the interdisciplinary team: a doctor, an ostomy nurse, a psychologist and a nutrition specialist. Many complications can be avoided by using an appropriately balanced diet, monitoring the nutritional status of the examined, and properly conducting nutritional education. A summary of dietary recommendations for patients with ostomies is presented in Table 2.

Researchers are particularly leaning toward appropriate stoma intervention and access to education for stoma patients, either in person or with the help of the Internet. However, there is still a lack of present good-quality nutritional research and supplementation guidelines for patients with an established intestinal stoma. Further research into an appropriate diet to minimize the risk of complications and re-hospitalization of patients with an intestinal stoma is needed.

## Figures and Tables

**Table 1:** Sites of absorption of nutrients in the intestine [15].

Part of the Gastrointestinal Tract	Absorbed Nutrients
Stomach	water, ethyl alcohol, copper, iodine, fluoride, molybdenum
Duodenum	calcium, phosphorus, magnesium, iron, copper, selenium, thiamin, riboflavin, niacin, biotin, folate, vitamins A, D, E, and K
Jejunum	lipids, monosaccharides, amino acids, small peptides, thiamin, riboflavin, niacin, pantothenate, biotin, folate, vitamin B_6_, vitamin C, vitamins A, D, E, and K, calcium, phosphorus, magnesium, iron, zinc, chromium, manganese, molybdenum,
Ileum	vitamin C, folate, vitamin B_12_, vitamin D, vitamin K, magnesium, others *, bile salts and acids
Large intestine	water, vitamin K, biotin, sodium, chloride, potassium, short-chain fatty acids

* Many additional nutrients may be absorbed from the ileum depending on transit time.

**Table 2:** Summary of nutritional recommendations for patients with ostomies.

Types of Ostomies	Summary of Nutrition Recommendations
Ileostomy	Avoiding products like popcorn, nuts, seeds, raisins, etc., as they can block stoma. Proper hydration is crucial to prevent excessive secretion and fluid disorders. Parenteral nutrition may be necessary due to the higher risk of nutritional deficiencies. Extending the diet after surgery should be done carefully and individually. This will allow easy detection and exclusion of harmful products for patients.
Colostomy	The diet does not deviate from the principles of a healthy and balanced diet recommended for healthy people. For possible constipation problems, plenty of exercise and a greater supply of food rich in insoluble fibre fractions, such as whole-grain bread, dark pasta and brown rice are recommended.
Both	The diet should be individually selected to meet the needs of the patient and be varied and rich in vitamins and minerals. The basis of the diet should be fresh products and foods and possibly without preservatives and artificial food dyes. The recommended form of food processing is cooking (traditional or steaming), possibly baking in foil and it is recommended to avoid fried, baked and stewed foods after frying. Products that should be avoided or consumed in limited quantities are alcohol (especially beer), sweets (especially chocolate and its products), carbohydrate drinks, egg yolk, salt and hot spices.

## Data Availability

Not applicable.
